# Venom-Derived Proteins from *Lonomia obliqua* Modulate Cytoskeletal Regulators and Inflammatory Responses in Human Chondrocytes

**DOI:** 10.3390/ijms27020934

**Published:** 2026-01-17

**Authors:** Miryam Paola Alvarez-Flores, Amanda Teixeira de Melo, Renata Nascimento Gomes, Thatiana Corrêa de Melo, Douglas Souza Oliveira, Marcelo Medina de Souza, Carlos DeOcesano-Pereira, Mauricio Barbugiani Goldfeder, Fernanda Faria, Ana Marisa Chudzinski-Tavassi

**Affiliations:** 1 Centre of Excellence in New Target Discovery (CENTD), Butantan Institute, São Paulo 05503-900, Brazil; a.melo.proppg@proppg.butantan.gov.br (A.T.d.M.); renata.gomes@fundacaobutantan.org.br (R.N.G.); thatiana.melo@butantan.gov.br (T.C.d.M.); douglas.oliveira@butantan.gov.br (D.S.O.); marcelo.msouza@alumni.usp.br (M.M.d.S.); carlos.ocesano@butantan.gov.br (C.D.-P.); 2 Center of Toxins, Immune-Response and Cell Signaling (CeTICS), Butantan Institute, São Paulo 05503-900, Brazil; 3 Development and Innovation Laboratory, Butantan Institute, São Paulo 05503-900, Brazil; mauricio.goldfeder@butantan.gov.br (M.B.G.); fernanda.faria@butantan.gov.br (F.F.)

**Keywords:** *Lonomia*, chondrocytes, IL-1β, inflammatory responses, cytokines, cytoskeletal pathways, Rac-1, RhoA, β-catenin

## Abstract

Osteoarthritis (OA) is a degenerative joint disease characterized by progressive cartilage loss, extracellular matrix degradation, chondrocyte apoptosis, and elevated inflammatory mediators. Chondrocytes respond to IL-1β and other inflammatory signals by secreting cytokines and activating transcriptional pathways that perpetuate inflammation. Because current therapies do not prevent OA progression, bioactive compounds with cytoprotective and immunomodulatory activity are of considerable interest. *Lonomia obliqua* bristle extract (LOCBE) and its recombinant proteins rLOPAP and rLOSAC exhibit cytoprotective, proliferative, and antioxidant effects in mammalian cells, as well as the ability to influence cytoskeletal dynamics. Given the importance of Rac-1, RhoA, Rab9, and β-catenin in chondrocyte function and cartilage homeostasis, we evaluated LOCBE, rLOPAP, and rLOSAC in human chondrocytes stimulated or not with IL-1β. LOCBE and rLOPAP induced IL-6 and IL-8 secretion, although at lower levels than IL-1β. LOCBE exerts a cytoprotective effect in IL-1β-treated chondrocytes and reduces β-catenin, RhoA, and Rab9 expression without affecting NF-κB p65 translocation. rLOPAP increased mitochondrial activity, cytokine secretion, Rab9 expression, and membrane-associated β-catenin, and under inflammatory conditions, enhanced Rac-1 levels. In contrast, rLOSAC did not induce inflammatory cytokines and decreased RhoA and Rac-1 expression while increasing membrane-associated β-catenin. These findings suggest that *L. obliqua* extract and its derived-proteins rLOPAP and rLOSAC modulate cytoskeletal regulatory pathways and inflammatory responses in chondrocytes, supporting their potential as therapeutic leads for targeting mechanisms relevant to OA progression.

## 1. Introduction

Osteoarthritis (OA) is characterized by the progressive degradation of extracellular matrix components, leading to degeneration of articular cartilage, increased levels of inflammatory mediators in the synovial fluid, such as interleukins and nitric oxide, and apoptosis of chondrocytes, the only cell type in cartilage [1,2,3,4]. In response to inflammatory stimuli, chondrocytes secrete cytokines, including interleukin-1β (IL-1β), Tumor necrosis factor alpha (TNF-α), interleukin 6 (IL-6), and interleukin 8 (IL-8), and undergo gene expression changes that sustain a self-amplifying inflammatory loop [5]. Therefore, the development of novel therapeutic approaches effective in suppressing OA progression is of great interest.

Animal venoms exhibit remarkable biochemical diversity and target multiple cellular pathways through distinct mechanisms [6,7]. Many animal venoms and secretions, including those from snakes, amphibians, and caterpillars, contain bioactive compounds capable of modulating inflammatory responses in joint cells such as chondrocytes [8,9]. These findings highlight the potential of venom-derived molecules as a source of novel therapeutic candidates for inflammatory joint diseases.

The bristle extract from the caterpillar *Lonomia obliqua* (LOCBE) contains two well-known proteins, LOSAC, a coagulation factor X activator, and LOPAP, a prothrombin activator. Beyond their pro-coagulant activity, both proteins display proliferative and cytoprotective effects in serum-deprived cells, including endothelial cells, fibroblasts, neutrophils, and neurons [10,11]. Recombinant LOSAC (rLOSAC) and LOPAP (rLOPAP) and derived peptides have been shown to increase fibroblast migration and type I collagen expression, promote in vivo wound healing by enhancing extracellular matrix (ECM) component expression and preserving cellular adhesion, and reduce reactive oxygen species formation, suggesting antioxidant activity [10]. Additionally, *L. obliqua* bristle extract modulates Ras-related C3 botulinum toxin substrate 1 (Rac-1) activation, indicating the presence of molecules capable of regulating cytoskeletal dynamics and cell migration [12]. 

The cytoskeleton is regulated by coordinated interactions among actin filaments, microtubules, intermediate filaments, and multiple signaling pathways [13]. Rac-1, a Rho family small GTPase, promotes actin polymerization and regulates cell–cell and cell–matrix adhesion, playing a key role in cell migration and tissue repair [14,15]. Persistent activation of Rac-1 and RhoA by IL-1β contributes to chronic inflammatory diseases, including OA [16,17]. Rac-1 regulates chondrocyte differentiation and proliferation [18], and aberrant activation has been linked to metalloproteinase 13 (MMP-13) induction and ECM degradation in OA [19,20]. Rac-1 inhibition prevents cartilage degeneration and ameliorates OA progression in animal models [16,20]. Nuclear factor-kappa B (NF-κB) and Wnt/β-catenin pathways, both activated by IL-1β [21], are central to OA-associated cartilage degradation [22]. Notably, Wnt/β-catenin signaling regulates actin cytoskeleton organization, and its inhibition improves OA in mice [23].

Here, we investigated the effects of LOCBE, rLOSAC, and rLOPAP on human chondrocytes exposed or not to IL-1β. LOCBE increased pro-inflammatory cytokine release (IL-6 and IL-8), albeit less strongly than IL-1β, and altered the expression of key cytoskeletal regulators, reducing β-catenin, RhoA, and Rab9, this last one under normal and inflammatory conditions, and without affecting NF-κB translocation. On the other hand, LOPAP did not affect NF-κB nuclear translocation, increased membrane-associated β-catenin, enhanced mitochondrial activity, induced IL-6 and IL-8 release, upregulated Rab9, and, under inflammatory stimulation, increased Rac-1 and membrane β-catenin. In contrast, rLOSAC did not induce inflammatory cytokines, and it decreased RhoA and Rac-1 expression and increased membrane-associated β-catenin. Taken together, these findings suggest that *L. obliqua* extract and its derived-proteins rLOPAP and rLOSAC differentially modulate cytoskeletal and inflammatory pathways associated with cartilage hypertrophy and the progression of osteoarthritis.

## 2. Results

### 2.1. Recombinant Proteins Preparation

rLOSAC and rLOPAP were purified according to previously established protocols (Figure S1) [24,25]. Endotoxins were removed, and the residual levels were determined for both proteins. The detected endotoxin levels for the recombinant proteins were <0.125 EU/mL for rLOSAC and ranged from 0.125 to 1.25 EU/mL for rLOPAP. In the context of biological research, 1 EU is commonly approximated to 100 picograms of endotoxin [26,27]. Based on this conversion, the endotoxin concentration in contact with the cells ranged from 12.5 to 125 pg/mL. While monocytes are highly sensitive and can be activated by very low LPS concentrations (2.5–5 pg/mL) to induce TNF-α and IL-6 production [28], the concentrations required to trigger significant cellular activation in other cell types, including chondrocytes, are typically in the ng/mL to µg/mL range [29,30].

### 2.2. LOCBE and Recombinant Proteins Are Not Toxic to Chondrocytes, and LOCBE Attenuates IL-1β–Induced Cytotoxic Effects

Chondrocytes were incubated for 24 h with different concentrations of LOCBE, rLOSAC, and rLOPAP to assess cell viability using the MTT assay. The MTT assay is a widely accepted method to evaluate cell viability and indirectly estimate the number of metabolically active cells [31]. In parallel, nuclear counts obtained by high-content screening (HCS) of Hoechst-stained nuclei were used to assess changes in total cell number.

**Figure 1:**
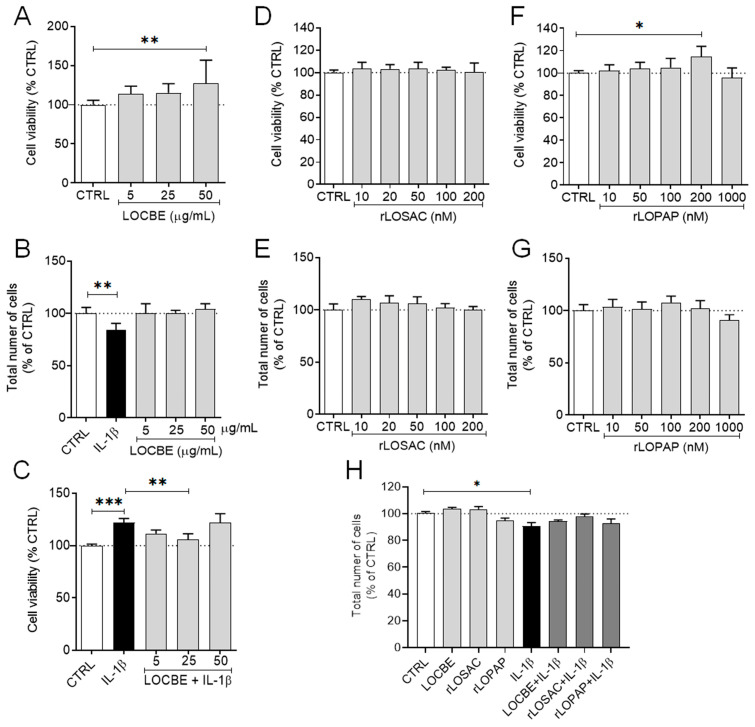
Effect of LOCBE and recombinant proteins on chondrocyte cell viability. Chondrocytes (1 × 10^4^ cells/well in 96-well plates) were incubated for 24 h with LOCBE, rLOSAC, and rLOPAP at different concentrations in DMEM-F12 medium containing 1% FBS. Cell viability was assessed by the MTT method (**A**,**C**,**D**,**F**). Cell counting was performed using HCS (**B**,**E**,**G**,**H**), as described in Section 4. Some treatments were conducted in the presence of an inflammatory stimulus using 1 ng/mL IL-1β (**C**,**H**). In H, LOCBE (50 µg/mL), rLOSAC (200 nM), and rLOPAP (200 nM) were used in the presence or absence of 1 ng/mL IL-1β. Data represent the mean ± SD from *n* = 9 independent replicates. * *p* < 0.05, ** *p* < 0.01, *** *p* < 0.001 (one-way ANOVA followed by Tukey’s test). CTRL, chondrocytes cultivated in basal DMEM-F12 medium containing 1% FBS (dotted line as a reference).

LOCBE and rLOPAP increased mitochondrial metabolic activity without affecting the total cell count (Figure 1A and Figure 1F, respectively; 100 ± 1.5%, 127.2 ± 9.9%, and 114.4 ± 3.9% for control (CTRL), 50 µg/mL LOCBE, and 200 nM rLOPAP, respectively; *n* = 9; * *p* < 0.05, ** *p* < 0.01 vs. control). On the other hand, rLOSAC did not alter mitochondrial metabolism or cell number (Figure 1D,E).

Treatment with 1 ng/mL IL-1β alone reduced the total cell number (Figure 1B) but increased mitochondrial activity (Figure 1C), consistent with mitochondrial stress previously reported in other cells under inflammatory conditions [32]. While LOCBE (25 µg/mL) alone did not affect cell number, in the presence of IL-1β, LOCBE attenuated the IL-1β-induced cell loss (Figure 1C). Neither LOCBE nor the recombinant proteins modified the IL-1β-mediated increase in metabolic activity.

Taken together, these results indicate that LOCBE, rLOPAP, and rLOSAC are not cytotoxic to chondrocytes. Furthermore, LOCBE can mitigate IL-1β-induced reduction in cell number, suggesting a potential cytoprotective effect under inflammatory conditions.

### 2.3. LOCBE, rLOPAP, or rLOSAC Do Not Affect NF-κB Translocation but Induce the Release of Inflammatory Cytokines

**Figure 2:**
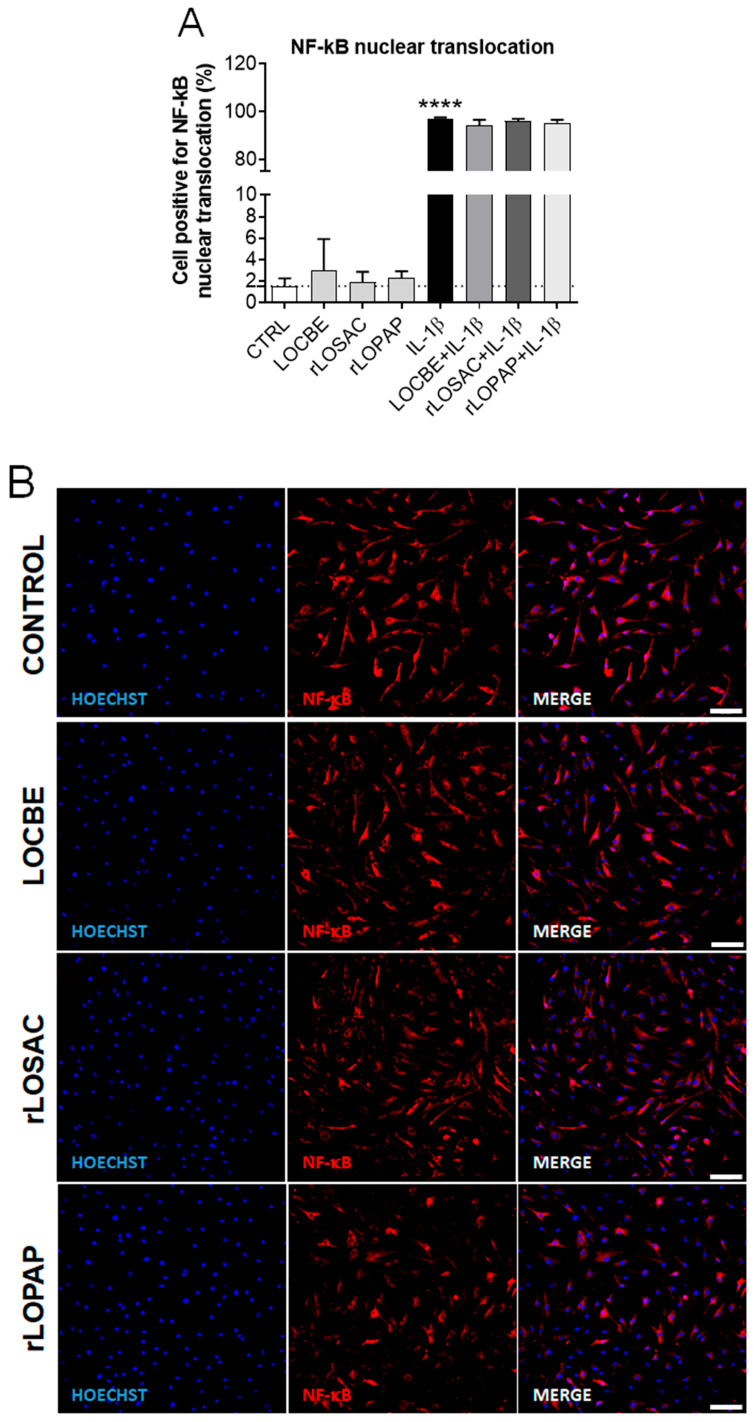

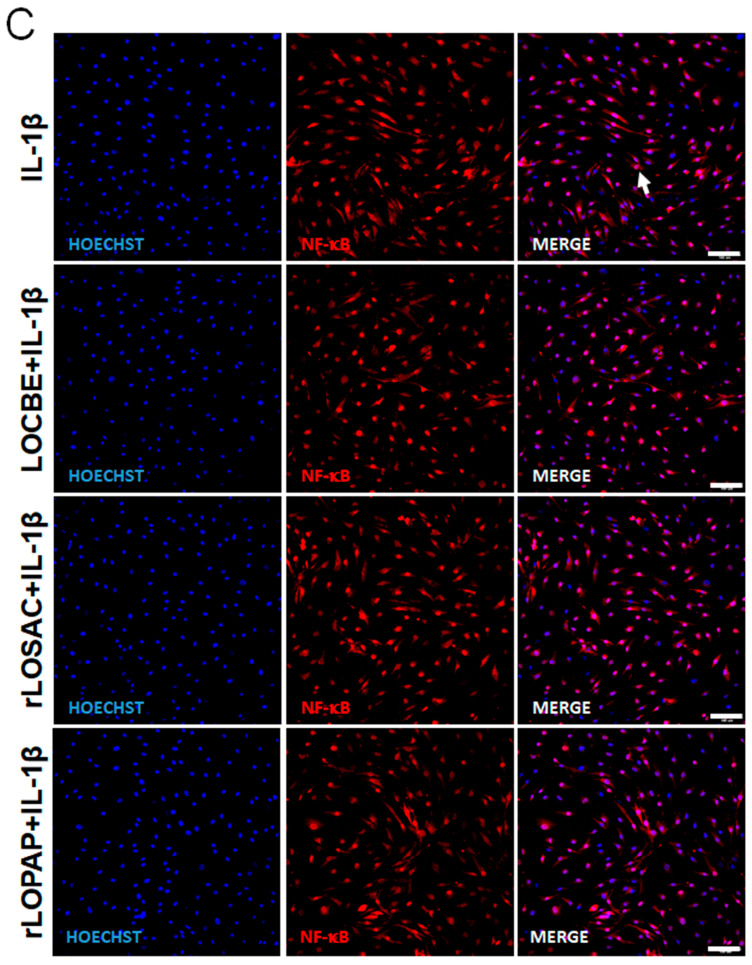
Nuclear translocation of NF-κB induced by LOCBE, rLOPAP, and rLOSAC. Chondrocytes (8 × 10^3^ cells/well in 96-well plate) were treated with LOCBE (50 µg/mL), rLOSAC (200 nM), and rLOPAP (200 nM), at the indicated concentrations for 30 min, or with 1 ng/mL IL-1β (positive control) for 30 min. In a separate set, LOCBE and proteins were incubated for 24 h before the addition of IL-1β for 30 min (treatment + IL-1β). Cells were fixed and immunolabeled with a primary antibody against NF-κB and an Alexa Fluor-647-conjugated secondary antibody (red), with nuclear counterstaining by Hoechst 33342 (blue). The stained samples were analyzed by HCS (20× magnification). (**A**) Data represent the mean ± SD from 16 different fields per well. **** *p* < 0.0001 (unpaired Student’s *t*-test, *n* = 3 independent replicates). CTRL, chondrocytes cultivated in basal DMEM-F12 medium containing 1% FBS (dotted line as a reference). Representative images of NF-κB translocation (red) and nuclei (blue) under basal (**B**) or inflammatory conditions (**C**). Control: untreated chondrocytes. The white arrow in the “IL-1β” panel indicates NF-κB translocated from the cytoplasm to the nucleus. Scale bar 100 μm.

It is well-established that IL-1β modulates inflammatory signaling pathways, which, in turn, induce the release of inflammatory cytokines that contribute to the activation and production of matrix-degrading proteases. IL-1β also induces the translocation of certain proteins, particularly the transcription factor NF-κB [21,33]. As a positive control, a 30 min treatment with 1 ng/mL IL-1β induced the nuclear translocation of NF-κB, as expected (Figure 2A–C). However, neither LOCBE nor rLOPAP or rLOSAC, when administered for 30 min, induced NF-κB translocation (Figure 2A,B). Chondrocytes were also pre-treated with LOCBE and the recombinant proteins for 24 h before a 30 min incubation with IL-1β. Under these conditions, none of the treatments was capable of reducing the IL-1β-mediated effect.

**Figure 3:**
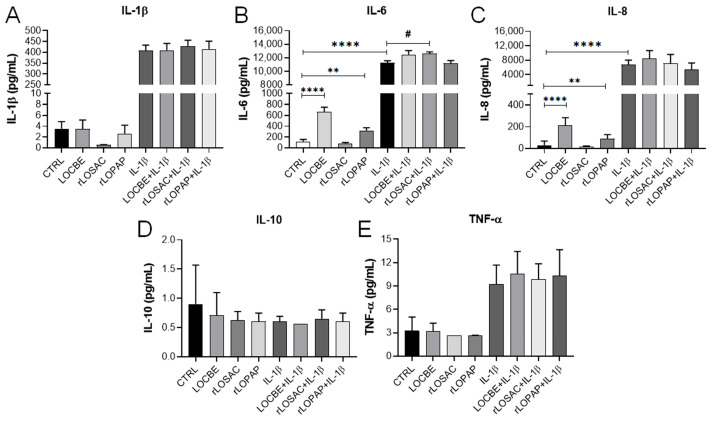
Effect of LOCBE, rLOPAP, and rLOSAC on cytokine release. Chondrocytes (1 × 10^4^ cells/well in 96-well plate), cultured in DMEM-F12 medium containing 1% FBS, were treated or not with 1 ng/mL IL-1β for 1 h. This was followed by the addition of 50 µg/mL LOCBE, 200 nM rLOSAC, or 200 nM rLOPAP and further 24 h incubation. Cell-free supernatants were collected, and cytokine levels were measured using a multiplex panel for IL-1β (**A**), IL-6 (**B**), IL-8 (**C**), IL-10 (**D**), and TNF-α (**E**). Data represent the mean ± SD from 16 different fields per well. ^#^ *p* > 0.05, ** *p* > 0.01, **** *p* > 0.0001 (one-way ANOVA followed by Tukey’s multiple-comparison post hoc test, *n* = 9 independent replicates). CTRL, chondrocytes cultivated in basal DMEM-F12 medium containing 1% FBS.

The subsequent experiment observed cytokine release after 24 h of treatment. As shown in Figure 3, 1 ng/mL IL-1β induced the release of IL-6, IL-8, TNF-α, and IL-1β (Figure 3A–C,E). Notably, IL-10 was not modulated by IL-1β in this cell model (Figure 3D). Interestingly, LOCBE and rLOPAP significantly induced the release of both IL-6 and IL-8 (Figure 3B and Figure 3C, respectively). For IL-6: 118.8 ± 37.8 pg/mL for control (CTRL); 667.3 ± 84.8 pg/mL for 50 µg/mL LOCBE; 319.7 ± 51.9 pg/mL for 200 nM rLOPAP; and 11,238 ± 329.1 pg/mL for 1 ng/mL IL-1β; *n* = 9; ** *p* < 0.01, **** *p* < 0.0001 vs. CTRL. Interestingly, 200 nM rLOSAC in the presence of IL-1β induced an increase in IL-6 (12,591 ± 271.4 pg/mL, ^#^ *p* < 0.05 vs. CTRL). For IL-8: 29.9 ± 12.9 pg/mL for control (CTRL); 211.8 ± 24.4 pg/mL for 50 µg/mL LOCBE; 91.6 ± 12.1 pg/mL for 200 nM rLOPAP; and 6803 ± 402.7 pg/mL for 1 ng/mL IL-1β; *n* = 9; ** *p* < 0.01, **** *p* < 0.0001 vs. CTRL.

Collectively, these results suggest that LOCBE contains compounds, including rLOPAP, that induce an inflammatory response, likely via signaling pathways other than NF-κB activation.

### 2.4. Morphological Changes Suggest Alterations in Cytoskeletal Proteins and Rac-1 Protein

**Figure 4:**
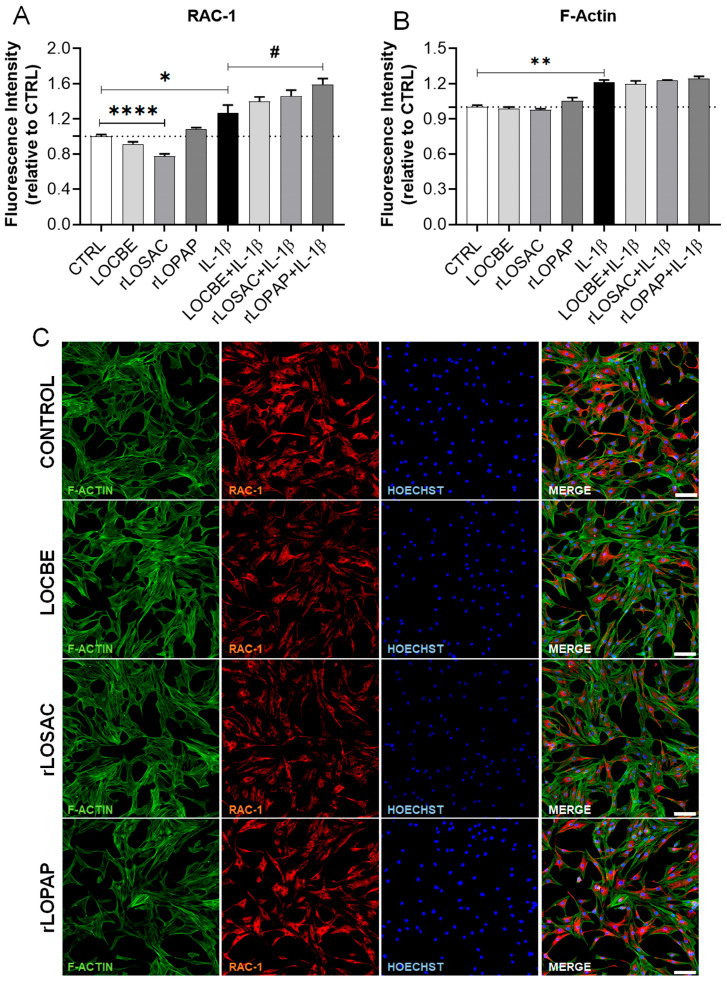

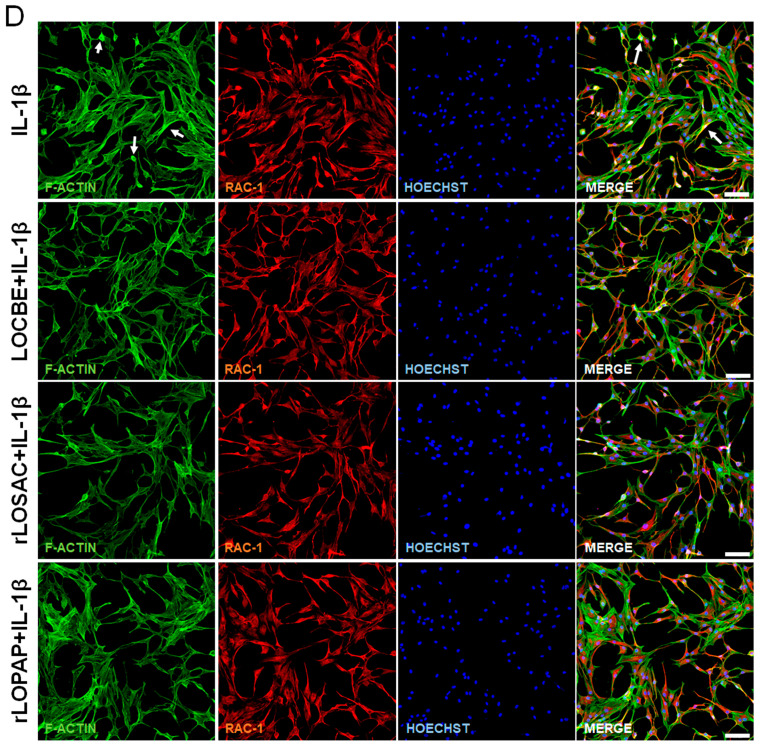
Effect of LOCBE, rLOPAP, and rLOSAC on Rac-1 and F-actin. Chondrocytes (8 × 10^3^ cells/well in 96-well plate) cultured in DMEM-F12 medium containing 1% FBS were treated or not with 1 ng/mL IL-1β for 1 h, followed by the addition of 50 µg/mL LOCBE, 200 nM rLOSAC, or 200 nM rLOPAP and incubation for 24 h. Cells were fixed and immunolabeled with a primary antibody against Rac-1 and an Alexa Fluor-647-conjugated secondary antibody (red) with nuclear counterstaining by Hoechst 33342 (blue) and F-actin staining by Alexa Fluor 488-phalloidin (green). The stained samples were analyzed by HCS (20× magnification) for quantification of Rac-1 (**A**) and F-actin (**B**). Data represent the mean ± SD from 16 different fields per well. ^#^ *p* > 0.05, * *p* > 0.05, ** *p* > 0.01, **** *p* > 0.0001 (unpaired Student’s *t*-test, *n* = 9 independent replicates). CTRL, chondrocytes cultivated in basal DMEM-F12 medium containing 1% FBS (dotted line as a reference). Representative images of F-actin (green), Rac-1 (red), and nuclei (blue) under basal (**C**) or inflammatory conditions (**D**). Control: untreated chondrocytes. The white arrow in the “IL-1β” panel highlights retracted and rounded cells, with yellow color indicating co-localization of Rac-1 and F-actin, an effect that was reduced in the presence of rLOPAP. Scale bar 100 μm.

We evaluated the fluorescence intensity of F-actin and the protein Rac-1 in chondrocytes treated with LOCBE, rLOPAP, or rLOSAC, in the presence or absence of IL-1β (Figure 4). Rac-1 is a small GTPase, a member of the Rho family of small GTPases, which is required for the regulation of the cytoskeleton, cell differentiation, and chondrogenesis [34]. Rac-1 has been implicated in various downstream functions, such as cell adhesion, proliferation, apoptosis, cell migration, and inflammatory responses, and, together with CDC42, is involved in the formation of lamellipodia and filopodia [35].

Under normal conditions, control cells exhibited predominantly cytoplasmic Rac-1 staining, appearing spread and less rigid (Figure 4C, Control panel). Conversely, in the presence of IL-1β, chondrocytes manifested significant morphological changes (Figure 4D, IL-1β panel). The cells became more spindle-shaped, and F-actin exhibited retracted stress fibers, appearing rounded with erratic migration and partially losing cell adhesion. This was accompanied by increased co-localization of Rac-1 with F-actin (Figure 4D, IL-1β panel). The effect of IL-1β significantly increased the quantified fluorescence intensity (F.I.) of both Rac-1 and F-actin (Figure 4A and Figure 4B, respectively): Rac-1: 1.0 ± 0.02 F.I. for control (CTRL) vs. 1.27 ± 0.09 F.I for 1 ng/mL IL-1β; *n* = 9 independent replicates; * *p* < 0.05, vs. CTRL; F-actin: 1.0 ± 0.03 F.I. for control (CTRL) vs. 1.21 ± 0.02 F.I for 1 ng/mL IL-1β; *n* = 9 independent replicates; ** *p* < 0.01 vs. CTRL.

Under normal conditions, rLOSAC was the only compound capable of significantly reducing the Rac-1 intensity (0.78 ± 0.03 F.I. for rLOSAC, *n* = 9 independent replicates; **** *p* < 0.0001, vs. CTRL) (Figure 4A), which resulted in less intense fluorescence compared to the control (Figure 4B). Morphologically, the rLOSAC-treated cells did not present drastic changes (Figure 4B, rLOSAC panel). Since Rac-1 is essential for chondrocyte differentiation and proliferation [34], the role of rLOSAC in these processes warrants deeper investigation, particularly because rLOSAC belongs to the hemolin family, which is implicated in cell differentiation and metamorphosis in Lepidoptera [36].

Interestingly, in the presence of IL-1β, rLOPAP increased Rac-1 protein expression compared to cells treated only with IL-1β (1.59 ± 0.07 F.I. for rLOPAP, *n* = 9 independent replicates; ^#^ *p* < 0.05, vs. IL-1β) (Figure 4A). However, rLOPAP also mitigated some of the morphological changes caused by IL-1β, such as the co-localization between Rac-1 and F-actin. This suggests that, under inflammatory conditions, rLOPAP may be modulating other key proteins that contribute to reducing the impact on the formation of the IL-1β-induced spindle-shaped cells.

### 2.5. Effect of LOCBE and Recombinant Proteins on the Expression of RhoA, β-Catenin and Rab-9

**Figure 5:**
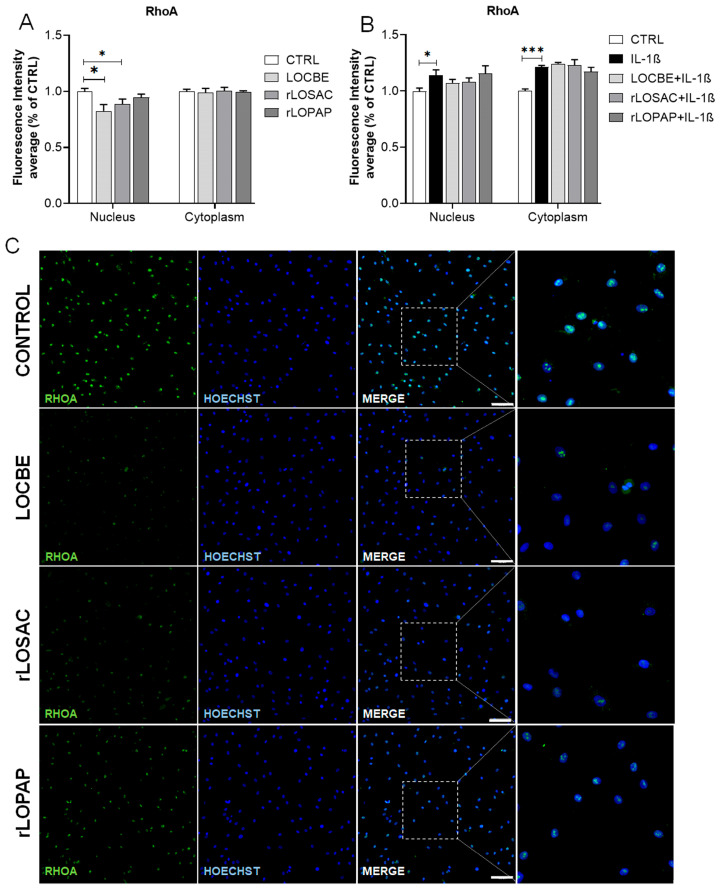

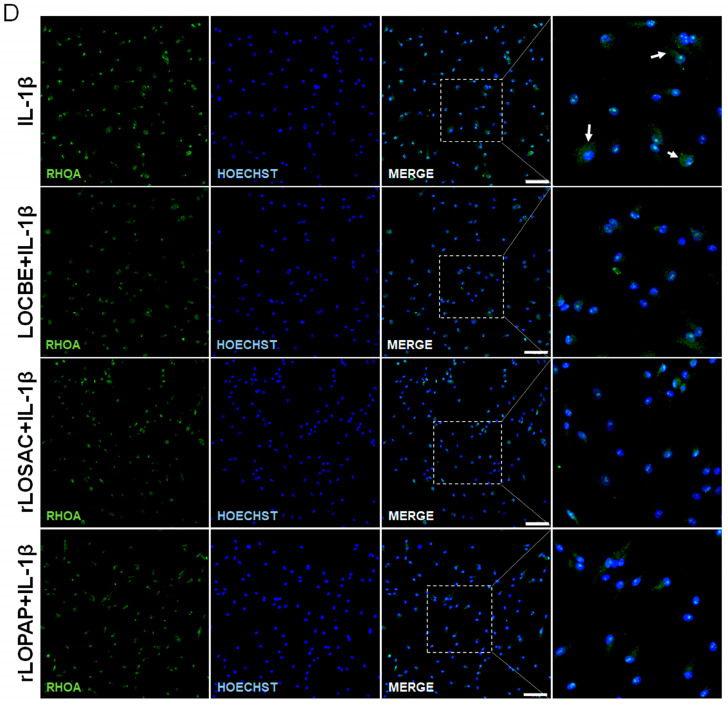
Effect of LOCBE, rLOPAP, and rLOSAC on RhoA. Chondrocytes (8 × 10^3^ cells/well in 96-well plate) cultured in DMEM-F12 medium containing 1% FBS were treated or not with 1 ng/mL IL-1β for 1 h, followed by the addition of 50 µg/mL LOCBE, 200 nM rLOSAC, or 200 nM rLOPAP and incubation for 24 h. Cells were fixed and immunolabeled with a primary antibody against RhoA and an Alexa Fluor-488-conjugated secondary antibody (green), with nuclear counterstaining by Hoechst 33342 (blue). The stained samples were analyzed by HCS (20× magnification), and fluorescence intensities were quantified in nuclear and cytoplasmic compartments under normal (**A**,**C**) and inflammatory (**B**,**D**) conditions. Data represent the mean ± SD from 16 different fields per well. * *p* > 0.05, *** *p* > 0.001 (unpaired Student’s *t*-test, *n* = 6 independent replicates). CTRL, chondrocytes cultivated in basal DMEM-F12 medium containing 1% FBS. Representative images of RhoA (green) and nuclei (blue) of chondrocytes under basal (**C**) or inflammatory conditions (**D**). Control: untreated chondrocytes. Zoomed-in panels highlight the more intense RhoA staining in the cytoplasm. The white arrow in the “IL-1β” zoomed-in panel indicates the presence of RhoA in the cytoplasm. Scale bar 100 μm.

In addition to Rac-1 activation, IL-1β-induced activation of RhoA has been associated with several types of chronic inflammatory disorders, including osteoarthritis (OA) [17]. Besides Rac-1, IL-1β was able to increase RhoA protein levels in both the nucleus and cytoplasm (Figure 5) with the following fluorescence intensity (F.I.): nucleus: 1.0 ± 0.03 for control (CTRL) vs. 1.14 ± 0.05 for 1 ng/mL IL-1β; *n* = 9 independent replicates; * *p* < 0.05, vs. CTRL; cytoplasm: 1.0 ± 0.02 for control (CTRL) vs. 1.21 ± 0.02 for 1 ng/mL IL-1β; *n* = 6 independent replicates; *** *p* < 0.001, vs. CTRL. Under these inflammatory conditions, neither LOCBE nor the recombinant proteins had any effect. However, under normal conditions (Figure 5B,D), both LOCBE and rLOSAC were able to reduce nuclear RhoA: 1.0 ± 0.02 F.I. for control (CTRL); 0.83 ± 0.06 F.I. for 50 µg/mL LOCBE; and 0.89 ± 0.05 F.I. for rLOSAC; *n* = 6 independent replicates; * *p* < 0.01 vs. CTRL.

**Figure 6:**
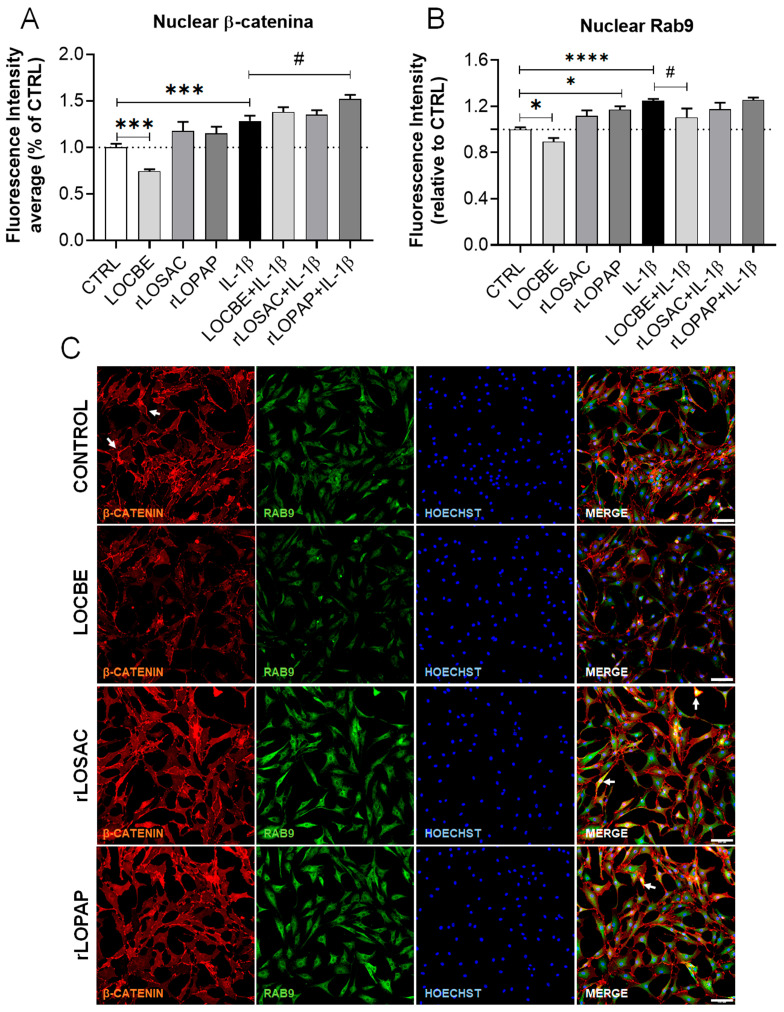

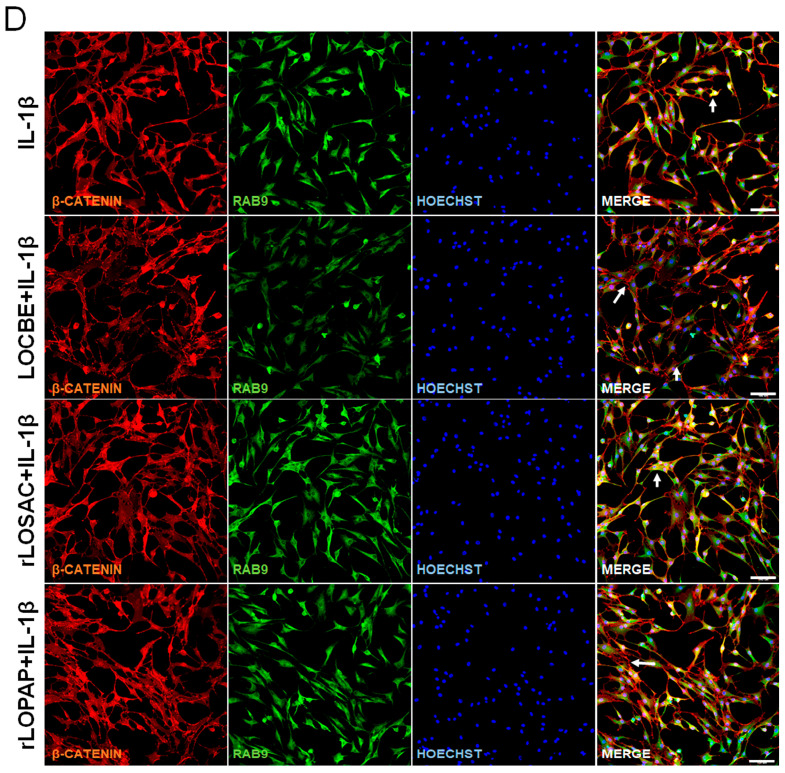
Effect of LOCBE, rLOPAP, and rLOSAC on β-catenin and Rab9. Chondrocytes (8 × 10^3^ cells/well in 96-well plate) cultured in DMEM-F12 medium containing 1% FBS were treated or not with 1 ng/mL IL-1β for 1 h, followed by the addition of 50 µg/mL LOCBE, 200 nM rLOSAC, or 200 nM rLOPAP and incubation for 24 h. Cells were fixed and immunolabeled with a primary antibody against β-catenin and an Alexa Fluor-647-conjugated secondary antibody (red), and a primary antibody against Rab9 with an Alexa Fluor-488-conjugated secondary antibody (green), with nuclear counterstaining by Hoechst 33342 (blue). The stained samples were analyzed by HCS (20× magnification) for quantification of β-catenin (**A**) and Rab9 (**B**). Data represent the mean ± SD from 16 different fields per well. ^#^ *p* > 0.05, * *p* > 0.05, *** *p* > 0.001, **** *p* > 0.0001 (unpaired Student’s *t*-test, *n* = 3 independent replicates). CTRL, chondrocytes cultivated in basal DMEM-F12 medium containing 1% FBS (dotted line as a reference). Representative images of β-catenin (red), Rab9 (green), and nuclei (blue) in chondrocytes under basal (**C**), or inflammatory conditions (**D**). Control: untreated chondrocytes. The white arrows in the “rLOSAC” and “rLOPAP” panels highlight retracted and rounded cells, with yellow color indicating the internalization of β-catenin. In samples treated with rLOSAC and rLOPAP, β-catenin is more intense at the cell junctions. Scale bar 100 μm.

This study also evaluated the effect of LOCBE and recombinant proteins on the Wnt/β-catenin signaling pathway, given its role in regulating actin cytoskeleton organization and its interaction with Rac-1 and RhoA proteins [37,38]. The inhibition of Wnt/β-catenin signaling has been shown to improve OA in mice [23]. We observed that IL-1β increased β-catenin nuclear translocation (Figure 6A–D), with fluorescence intensity (F.I.) values of 1.0 ± 0.04 for control (CTRL) versus 1.28 ± 0.06 for IL-1β (*n* = 3 independent replicates; * *p* < 0.001). Under basal conditions (Figure 6C), LOCBE was the only treatment that reduced β-catenin expression (0.74 ± 0.02 F.I. for LOCBE; *n* = 3; * *p* < 0.001 vs. CTRL). Under inflammatory conditions (Figure 6A–D), rLOPAP increased β-catenin expression (1.52 ± 0.04 F.I. for rLOPAP in the presence of IL-1β; *n* = 3; *p* < 0.05 vs. IL-1β alone).

In this study, we observed that Rab9 protein levels were increased by IL-1β (Figure 6B), with a fluorescence intensity (F.I.) of 1.0 ± 0.02 for control (CTRL) versus 1.25 ± 0.01 for IL-1β (*n* = 3 independent replicates; **** *p* < 0.0001). Conversely, LOCBE was able to mitigate the effect of IL-1β, reducing Rab9 levels both under basal conditions (0.89 ± 0.03 F.I. vs. CTRL; *n* = 6; * *p* < 0.05) and under inflammatory conditions (1.25 ± 0.01 F.I. for IL-1β vs. 1.1 ± 0.08 F.I. for LOCBE/IL-1β; *n* = 3; ^#^ *p* < 0.05) (Figure 6B–D). rLOSAC did not modulate Rab9, whereas rLOPAP increased Rab9 under basal conditions (1.17 ± 0.03 F.I. vs. CTRL; *n* = 3; * *p* < 0.05).

Interestingly, differences in the β-catenin staining pattern at the plasma membrane of chondrocytes treated with rLOSAC or rLOPAP were observed (Figure 6). Figure 6C distinguishes the cytoplasmic localization of Rab9. In the presence of rLOSAC and rLOPAP, the formation of retracted and rounded cells is evident, with yellow color (in merged channels) indicating the cytoplasmic localization of a portion of β-catenin, as indicated by white arrows. Furthermore, in samples treated with rLOSAC and rLOPAP, β-catenin appears more intense at the cell junctions. Under inflammatory conditions (Figure 6D), the reduction in Rab9 and nuclear β-catenin induced by LOCBE is clear, as is the accumulation of β-catenin at cell junctions in chondrocytes treated with rLOPAP.

These data reinforce the hypothesis that LOCBE and its derived proteins modulate members of the Rho GTPase family and the Wnt/β-catenin signaling pathway, which could impact cytoskeletal dynamics and extracellular matrix organization.

## 3. Discussion

The caterpillar *Lonomia obliqua* is well-known for causing a hemorrhagic syndrome and a systemic pro-inflammatory response in patients who come into contact with its urticating bristles [10,39]. In recent years, studies have been conducted to elucidate the molecular mechanisms underlying the envenomation process. Some studies have indicated that LOCBE modulates cell migration, cell adhesion, and cytoskeletal dynamics [10,12]. Two well-characterized anti-apoptotic proteins derived from LOCBE, namely LOPAP and LOSAC, are responsible for some of these observed effects [10,11]. In this study, we investigated the direct effects of LOCBE and the recombinant proteins rLOPAP and rLOSAC on human chondrocytes, both with and without IL-1β exposure. We assessed their impact on cytokine release, NF-κB nuclear translocation, and the expression of key cytoskeletal regulators, including Rac-1, RhoA, Rab9, and β-catenin.

The cytoskeleton is regulated by coordinated interactions among actin filaments, microtubules, intermediate filaments, and multiple signaling pathways [13]. In this study, we observed that LOCBE also modulates mitochondrial metabolism (Figure 1A,B). These cytoskeletal reorganization processes are closely linked to mitochondrial function. For instance, actin filaments can activate glycolytic enzymes through direct binding, thereby regulating mitochondrial metabolism [40].

Our results demonstrate that LOCBE was not toxic to chondrocytes, as it did not modulate cell number, but it increased mitochondrial metabolism (Figure 1A,B). In contrast, IL-1β reduced the cell number and increased mitochondrial metabolism (Figure 1B,C). It is well-established that IL-1β can induce cell death through mitochondrial dysfunction [32]. Under these inflammatory conditions, LOCBE was able to counteract the effect of IL-1β on mitochondrial metabolism (Figure 1C). This could result from an increase in mitochondrial mass or the modulation of mitochondrial dehydrogenases, as has been observed in other systems [41]. Furthermore, in some cellular models, LOCBE increases cell viability [10,11,42,43], an effect that may be attributed to cytoprotective and anti-apoptotic proteins present in the venom [10]. We also assayed two well-known cytoprotective molecules derived from LOCBE, rLOPAP, and rLOSAC, in chondrocytes. Although neither rLOSAC nor rLOPAP reversed the effect of IL-1β (Figure 1H), rLOPAP increased mitochondrial metabolism under basal conditions (Figure 1F). This finding is consistent with previous literature reporting that LOCBE and rLOPAP enhance mitochondrial metabolism [10].

Regarding the NF-κB pathway, neither LOCBE nor the recombinant proteins modulated NF-κB translocation in chondrocytes (Figure 2). This result differs from a previous study in which LOCBE showed a potent pro-inflammatory effect by activating the NF-κB pathway in macrophages [44]. This cell-type-specific effect is particularly interesting, given that LOCBE and rLOPAP induced the release of IL-6 and IL-8 in chondrocytes (Figure 3). NF-κB is a pivotal mediator of inflammatory responses and regulates multiple aspects of innate and adaptive immunity, playing a major role in inflammatory diseases such as rheumatoid arthritis [45].

Concerning cytokine release, it has been previously demonstrated that *Lonomia obliqua* caterpillar extract induces an inflammatory profile in fibroblasts, promoting the release of IL-6 and IL-8 [46], similar to our observations in chondrocytes treated with LOCBE and rLOPAP (Figure 3). Furthermore, LOPAP has been suggested to contribute to the extract’s pro-inflammatory effect, as it promotes the production of prostacyclin (PGI_2_) and nitric oxide in HUVECs [47]. However, no cytokine release was observed in neutrophils or rat microvascular endothelial cells [48], highlighting the cell type dependence of this response.

Several signaling pathways can lead to increased IL-6 release independently of the canonical NF-κB pathway. For instance, the p38 MAPK and ERK1/2 pathways [49], or the non-canonical NF-κB activation via Notch, involving p53 and IKKα/IKKβ [50], are potential mechanisms. Therefore, further investigation is required to determine the role of these pathways in the pro-inflammatory effects of LOCBE and rLOPAP in chondrocytes.

Another pathway associated with both actin cytoskeleton organization and IL-6 regulation is the Wnt/β–catenin pathway [21]. The activation of both the NF-κB and Wnt/β-catenin pathways by IL-1β plays a central role in OA-associated cartilage degradation [21,22]. A complex interaction exists between the Wnt/β-catenin and Rho GTPase pathways, which regulate differentiation processes, such as hypertrophy-like changes, in the pathogenesis of OA. This interplay makes them promising targets for new therapeutic development [37]. Notably, the inhibition of the Wnt/β–catenin pathway has been shown to ameliorate OA in mice [23].

Literature data demonstrate that *Lonomia obliqua* extract and its derived compounds can regulate cell adhesion, increase fibroblast migration and type I collagen expression, promote in vivo wound healing by enhancing ECM component expression, and reduce reactive oxygen species formation, suggesting an antioxidant activity [10]. Furthermore, they regulate cytoskeletal dynamics and cell migration through the activation of Rac-1 [12]. Rac-1 belongs to the Rho family of small GTPases, which is implicated in cytoskeleton regulation, cell differentiation, and chondrogenesis [34]. An imbalance in Rho GTPase signaling is associated with numerous pathologies [51]. In our study, morphological changes were observed in IL-1β-treated chondrocytes, including the translocation and increased expression of Rac-1 (Figure 4). Rac-1 primarily regulates the actin cytoskeleton [15], and we confirmed its colocalization with F-actin (Figure 4). The persistent activation of both Rac-1 and RhoA by IL-1β contributes to chronic inflammatory diseases, including osteoarthritis (OA) [16,17].

Dysregulation of RhoA and its downstream effector ROCK (the RhoA/ROCK pathway) has been associated with actin cytoskeleton modifications, β-catenin imbalance, stress fiber formation, the emergence of spindle-shaped cells, and the promotion of chondrocyte hypertrophy. These processes collectively contribute to the chronic inflammatory profile in OA [16,17,52,53]. The reduction in nuclear RhoA by LOCBE and rLOSAC may mitigate the effects of RhoA, such as activation of transcription factors [54] and chondrocyte dedifferentiation [46]. This, in turn, could lead to reduced cartilage degradation and hypertrophy [16,17].

The data presented reflect the up- or downregulation of the expression and translocation of the studied proteins. However, the biological significance of these modulations remains to be further evaluated, as certain molecules require activation to function. This is the case for Rac-1 and RhoA, two small GTPases whose activity depends on being bound to GTP (active state) rather than GDP (inactive state) [14,15].

Our results demonstrate that LOCBE, rLOSAC, and rLOPAP differentially modulate the proteins Rac-1, RhoA, β-catenin, and Rab9. LOCBE increases inflammatory cytokines, reduces β-catenin and RhoA, and also counteracts the IL-1β-induced increase in Rab9. This suggests that the crude extract contains components with both pro-inflammatory effects and others that may protect against cartilage degradation by mitigating specific effects of IL-1β, particularly the upregulation of Rab9. Rab9 is a small GTPase protein involved in multiple intracellular trafficking pathways, facilitating vesicular transport between the trans-Golgi network (TGN), late endosomes, and lysosomes. It fulfills critical cellular roles, such as lipid transport and participation in lysosomal biogenesis [55]. Recent literature on OA discusses the role of other Rab small GTPases in inhibiting autophagy and accelerating OA progression [56]; however, prior to this study, the specific role of Rab9 in OA had not been reported. Regarding rLOSAC, it reduces Rac-1 and RhoA levels without affecting Rab9, indicating a potentially beneficial effect on chondrocytes. In contrast, rLOPAP increases inflammatory cytokines, Rac-1, and Rab9, has no effect on RhoA, and promotes the accumulation of β-catenin in the nucleus and at plasma membrane junctions, both under basal and inflammatory conditions (Figure 5 and Figure 6). This suggests a mechanism distinct from rLOSAC, with a greater propensity to induce cell differentiation. The activation of β-catenin stimulates articular matrix catabolism and excessive remodeling, processes associated with the loss of the chondrocyte phenotype [57].

A characteristic of early OA stages is the hypertrophy of articular chondrocytes, which leads to significant cytoskeletal remodeling [58]. Therefore, molecules capable of regulating these alterations are of great interest, as it has been suggested that inhibiting the mechanisms leading to cartilage hypertrophy could be a therapeutic target to reduce OA progression [59]. Given that the dysregulation of Rho GTPases is a hallmark of OA progression and chondrocyte dedifferentiation, the ability of rLOSAC to stabilize these pathways in vitro provides a strong rationale for future in vivo studies. Intra-articular administration of this protein could potentially offer a targeted approach to preserve cartilage structural integrity and stabilize the chondrocyte phenotype, minimizing the systemic inflammatory risks associated with the crude extract. In this context, the components of the *Lonomia obliqua* extract are highly interesting candidates for further investigation of their potential as modulators of cellular targets associated with cartilage hypertrophy and osteoarthritis progression.

## 4. Materials and Methods

### 4.1. Venom and Proteins Preparation

*Lonomia obliqua* crude bristle extract (LOCBE) was kindly provided by the Butantan Institute. The bristles extract was prepared from caterpillars obtained from southern Brazil as previously described [44]. The extract was homogenized in phosphate-buffered saline (PBS, pH 7.4), and its protein concentration was determined using the Pierce BCA Protein Assay Kit (Thermo Scientific #23225, Rockford, IL, USA), according to the manufacturer’s protocol. The resulting extract was stored at −80 °C until use. rLOSAC and rLOPAP proteins were produced in competent *Escherichia coli* BL21 (DE3) cells. Purification and subsequent preparation of these proteins were carried out as previously reported [24,25]. Endotoxins were removed using the Pierce™ High-Capacity Endotoxin Removal Spin Columns (Thermo Scientific #88277, Rockford, IL, USA). Residual endotoxin levels were subsequently quantified using a Limulus Amebocyte Lysate (LAL) gel-clot assay according to the manufacturer’s instructions (LONZA #N294-125, Walkersville, MD, USA).

In the present study, the ranges of LOCBE, rLOSAC, and rLOPAP concentrations were selected based on previous in vitro biological findings from our group or from the literature [10,11,12,44].

### 4.2. Cell Culture

Human primary knee articular chondrocytes (NHAC-kn) were purchased from Lonza (#CC-2550, Lot 8F3336, Walkersville, MD, USA) and cultured in a humidified incubator at 37 °C with 5% CO_2_, according to the manufacturer’s instructions. Cells were initially maintained in CBM Basal Medium (Lonza #CC-3217, Walkersville, MD, USA) supplemented with CGM SingleQuots™ (Lonza #CC-3217), recommended for chondrocyte growth, and cryopreserved at passage 5. Experiments were performed with NHAC-kn cells (up to passage 6) cultured in DMEM/F-12 medium (Gibco #11320033, Bleiswijk, The Netherlands) supplemented with 1% fetal bovine serum (FBS) (Gibco #12657029, Sao Paulo, SP, Brazil).

### 4.3. Chondrocyte Viability Assay

Chondrocyte viability was assessed using the MTT assay, a colorimetric method based on the reduction in tetrazolium salt (MTT) to insoluble formazan crystals by mitochondrial dehydrogenases, predominantly succinate dehydrogenase [60]. In this work, the MTT assay was used to analyze mitochondrial metabolism based on the reduction in tetrazolium salt by mitochondrial dehydrogenases [41].

Chondrocytes (1 × 10^4^ cells/well) were seeded in 96-well plates. Following cell adherence, chondrocytes were treated with LOCBE, rLOSAC, or rLOPAP and incubated for 24 h at 37 °C with 5% CO_2_ in DMEM/F-12 containing 1% FBS. After treatment, the culture medium was removed and replaced with 100 µL of MTT solution (0.5 mg/mL thiazolyl blue tetrazolium bromide in PBS; Sigma-Aldrich #M5655, St. Louis, MO, USA) and incubated for 3 h. The MTT solution was then removed, and 100 µL of dimethyl sufoxide (DMSO) (Sigma-Aldrich #D8418, St. Louis, MO, USA) was added to solubilize the resultant formazan crystals. Absorbance was measured at 540 nm using a SpectraMax 190 spectrophotometer (Molecular Devices, San Jose, CA, USA). Cell viability was expressed as a percentage relative to the untreated control group (CTRL) cultured in DMEM/F-12 containing 1% FBS, which was set to 100%.

### 4.4. Cytokine Release Analysis

Chondrocytes (1 × 10^4^ cells/well) were seeded in 96-well plates. Following cell adherence, chondrocytes were treated with 50 µg/mL LOCBE, 200 nM rLOSAC, or 200 nM rLOPAP and incubated for 24 h at 37 °C with 5% CO_2_ in DMEM/F-12 containing 1% FBS. After treatment, cell-free supernatants were collected for cytokine quantification. Levels of IL-6, IL-8, TNF-α, IL-10, and IL-1β were measured using the MILLIPLEX^®^ MAP Human Cytokine/Chemokine Magnetic Bead Panel (Millipore #HCYTOMAG-60K-05, Burlington, MA, USA), following the manufacturer’s instructions. Data acquisition was performed with Luminex xPONENT^®^ 4.3 software, and analysis was conducted using MILLIPLEX^®^ Analyst 5.1. Cytokine concentrations are reported as pg/mL.

### 4.5. Immunofluorescence, Acquisition, and Image-Based Analysis

Chondrocytes (8 × 10^3^ cells/well) were seeded in 96-well microplates (Greiner Bio-One #655986, Advanced, Frickenhausen, Germany) using DMEM-F12 medium containing 10% FBS and incubated for 48 h to allow for complete cell adherence, at 37 °C with 5% CO_2_. The FBS concentration was subsequently reduced to 1% for all treatment periods.

For the analysis of phospho-NF-κB p65 (rabbit monoclonal, Cell Signaling #3033S, Danvers, MA, USA) translocation, cells were treated with 50 µg/mL LOCBE, 200 nM rLOPAP, or 200 nM rLOSAC for 30 min. Alternatively, cells were pre-incubated with the aforementioned compounds for 24 h prior to stimulation with 1 ng/mL of recombinant human IL-1β (R&D Systems #201-LB-025, Minneapolis, MN, USA) for an additional 30 min. As a positive control for translocation, chondrocytes were incubated with 1 ng/mL IL-1β for 30 min. For the analysis of intracellular Rab9 (mouse monoclonal, Invitrogen #MA3-067, Carlsbad, CA, USA), β-catenin (rabbit monoclonal, Cell Signaling #8480, Danvers, MA, USA), RhoA (mouse monoclonal, Santa Cruz Biotechnology #sc-418, Dallas, TX, USA), and Rac-1 (rabbit polyclonal, Invitrogen #PA1-091-A) expression, cells were treated with 50 µg/mL LOCBE, 200 nM rLOPAP, or 200 nM rLOSAC for 24 h.

#### 4.5.1. Immunofluorescence Staining Protocol

Following treatment, cells were washed with PHEM buffer (60 mM PIPES, 25 mM HEPES, 10 mM EGTA, 2 mM MgCl_2_, pH 6.9) and fixed in cold 4% paraformaldehyde for 1 h. Permeabilization was carried out using 0.1% Triton X-100 for 5 min, followed by a blocking step with 1% bovine serum albumin (BSA) for 30 min. Primary antibodies were diluted in PHEM buffer containing 1% BSA as follow: NF-κB p65 (1:500), Rab9 (1:100), RhoA (1:50), β-catenin (1:100), and Rac-1 (1:100), and incubated overnight at 4 °C. After three washes with PHEM-glycine buffer (PHEM containing 100 mM glycine), nuclei were stained with Hoechst 33342 (1/1000, Invitrogen #H3570) and secondary antibodies: Alexa Fluor 488 goat anti-mouse IgG (1:1000, Invitrogen #A11029, Carlsbad, CA, USA) or Alexa Fluor 647 goat anti-rabbit IgG (1:1000, Invitrogen #A21244, Carlsbad, CA, USA) for 1 h at room temperature. F-actin was counterstained with Alexa Fluor 488-Phalloidin (1:3000; Invitrogen #A12379, Carlsbad, CA, USA).

#### 4.5.2. Image Acquisition, Analysis, and Quantification

Images were acquired using the ImageXpress^®^ Micro Confocal High-Content Imaging System (Molecular Devices, San Jose, CA, USA). A total of 16 fields per well were acquired at 20× magnification. Image Analysis was subsequently performed using the MetaXpress^®^ Acquisition & Analysis Software version 6.7.2.290 (Molecular Devices, San Jose, CA, USA). Data obtained by HCS represent fluorescence intensity relative to secondary-only controls.

Cell quantification based on images was performed according to the following steps: Nuclear segmentation: Nuclei were automatically identified within illumination-corrected Hoechst-33342 to define nuclear boundaries. Specifically, the fluorescence intensity above the local background was used for nucleus detection.

Cytoplasm segmentation: An internal mask (representing the cytoplasm) was defined by dilating the nuclear mask outward to the edge of the Hoechst-33342 signal. The intensity above the local background in the stained area was used to distinguish positive from negative cells.

NF-κB nuclear translocation: This was calculated as the ratio of nuclear to cytoplasmic fluorescence intensity (inner/outer intensity ratio), with a correlation coefficient ≥ 0.6 used for object selection.

Protein expression: Rab9, RhoA, and Rac-1 intensities were quantified in both nuclear and cytoplasmic compartments, or as the total average intensity.

β-catenin quantification: The mean fluorescence intensity was quantified using CellProfiler™ version 4.2.5 (www.cellprofiler.org).

### 4.6. Statistical Analysis

All data analyses were performed using at least three independent biological experiments, with *n* representing the number of independent replicates as indicated in the figure legends, unless otherwise specified. Data are expressed as mean ± standard deviation (SD). Group comparisons were performed using unpaired Student’s *t*-tests or one-way ANOVA followed by Tukey’s multiple-comparison post hoc tests, as appropriate, using GraphPad Prism version 8.02 (GraphPad Software, La Jolla, CA, USA). Statistical significance was defined as *p* ≤ 0.05.

## 5. Conclusions

In conclusion, this study provides evidence that *Lonomia obliqua* caterpillar bristle extract (LOCBE) and its recombinant proteins, rLOSAC and rLOPAP, differentially modulate key pathways in human chondrocytes that are critically involved in osteoarthritis (OA) pathogenesis. While LOCBE and rLOPAP exhibit a pro-inflammatory capacity by inducing IL-6 and IL-8 release, they also demonstrate distinct cytoprotective and modulatory properties. LOCBE, notably, reduced the expression of β-catenin, RhoA, and the IL-1β-induced upregulation of Rab9, suggesting a capacity to counteract specific degenerative processes. Conversely, rLOSAC presented a more uniformly beneficial profile by reducing pro-hypertrophic mediators Rac-1 and RhoA without inducing inflammation, positioning it as a promising anti-OA candidate. The finding that these effects occurred independently of NF-κB translocation underscores the involvement of alternative signaling pathways. The dual nature of LOCBE, containing both inflammatory and protective components, highlights the complex bioactivity of this venom and its potential as a rich source of biological leads. These findings strongly support the further investigation of these molecules, particularly rLOSAC, as novel compounds targeting cytoskeletal dynamics and inflammatory responses, providing a strong rationale for future in vivo studies in osteoarthritis.

## Data Availability

The original contributions presented in this study are included in the article/Supplementary Material. Further inquiries can be directed to the corresponding author.

## Supplementary Materials

The following supporting information can be downloaded at: https://www.mdpi.com/article/10.3390/ijms27020934/s1.
