# Supplementary material for: Venom-Derived Proteins from Lonomia obliqua Modulate Cytoskeletal Regulators and Inflammatory Responses in Human Chondrocytes
Source: Int J Mol Sci. 2026 Jan 17;27(2):934. doi: 10.3390/ijms27020934 (PMC12841748; doi:10.3390/ijms27020934)
Supplement: Supplementary file 1 [file ijms-27-00934-s001.zip › ijms-4006888-supplementary.pdf]

Supplementary Material

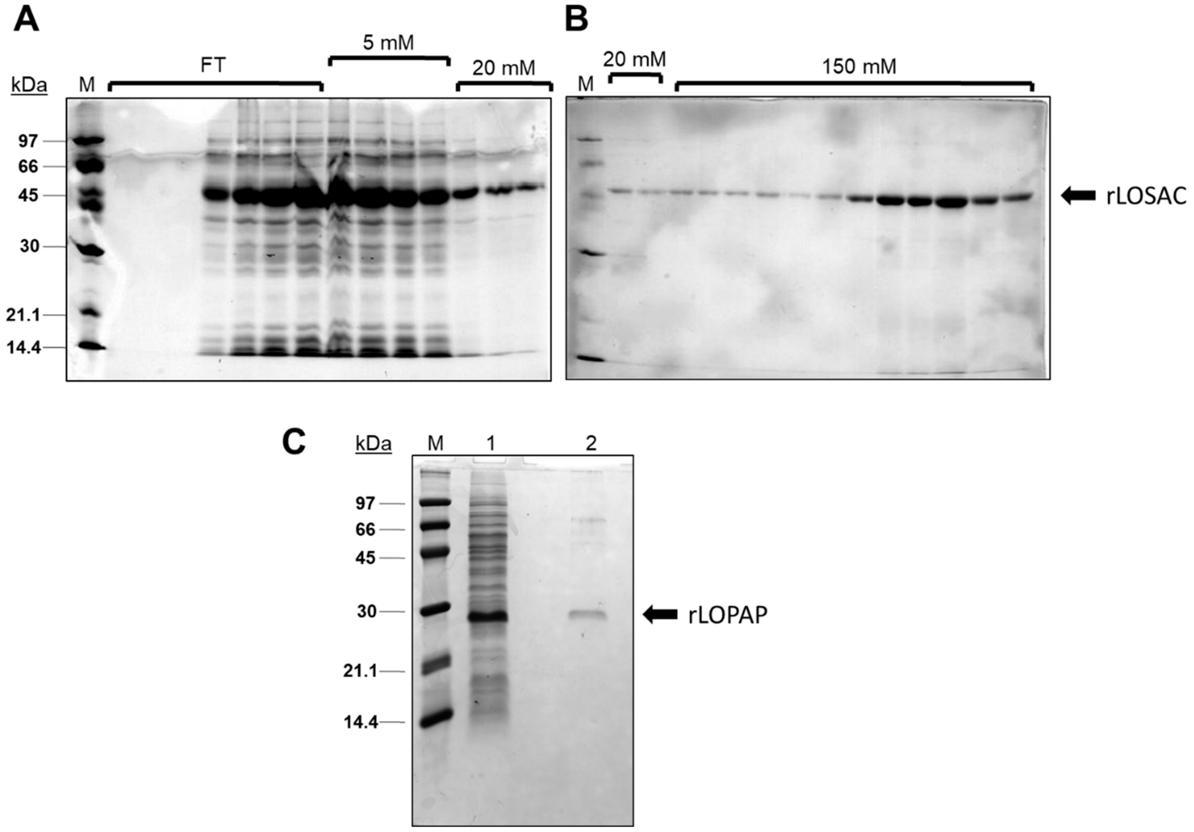

FIGURE **S1. Purification of recombinant LOSAC and LOPAP.** rLOPAP and rLOSAC were expressed in *E. coli* BL21(DE3) according to Reis et al. [24] and Alvarez-Flores et al. [25], respectively. Inclusion bodies were solubilized in 8 M urea, refolded, and purified by Ni^{2+}-chelating affinity chromatography. Fractions were analyzed by 12.5% SDS-PAGE under reducing conditions. **(A, B) Analysis of purified LOSAC:** (A) flow-through (FT) after sample loading and fractions eluted with buffer containing 5 mM and 20 mM imidazole. (B) Additional fractions eluted with 20 mM and 150 mM imidazole. **(C) Analysis of purified LOPAP.** Line 1: The recombinant LOPAP was primarily found within inclusion bodies. Lane 2 represents the fraction obtained after elution from the Ni^{2+}-Sepharose column. In all gels, "M" represents 10 µL of Low Molecular Weight SDS Marker Kit (Cytiva #17044601, Marlborough, MA, USA).
